# Impact of babyface schema on time perception: Insights from neutral and crying facial expressions

**DOI:** 10.1002/pchj.766

**Published:** 2024-06-03

**Authors:** Lina Jia, Bingjie Shao, Lili Wang, Xiaocheng Wang, Zhuanghua Shi

**Affiliations:** ^1^ School of Education Jiangnan University Wuxi China; ^2^ School of Educational Science Huaiyin Normal University Huaian China; ^3^ Department of Psychology Ludwig‐Maximilians University Munich Munich Germany

**Keywords:** attention, duration perception, embodiment, infant facial expression, sex difference

## Abstract

Facial expressions in infants have been noted to create a spatial attention bias when compared with adult faces. Yet, there is limited understanding of how adults perceive the timing of infant facial expressions. To investigate this, we used both infant and adult facial expressions in a temporal bisection task. In Experiment 1, we compared duration judgments of neutral infant and adult faces. The results revealed that participants felt that neutral infant faces lasted for a shorter time than neutral adult faces, independent of participant sex. Experiment 2 employed sad (crying) facial expressions. Here, the female participants perceived that the infants' faces were displayed for a longer duration than the adults' faces, whereas this distinction was not evident among the male participants. These findings highlight the influence of the babyface schema on time perception, nuanced by emotional context and sex‐based individual variances.

## INTRODUCTION

The way we perceive time is crucial to our daily existence. We have all had moments when time seems to stretch out, particularly in the face of danger, such as when a snake is about to strike. This phenomenon has been extensively researched (Droit‐Volet, 2019; Droit‐Volet et al., 2004; Droit‐Volet et al., 2013; Fayolle et al., 2015; Gil & Droit‐Volet, 2011). The lengthened time experienced in response to threats can help us react swiftly and be prepared for action (Droit‐Volet, 2019; Droit‐Volet et al., 2013). The distortion of our perception of time in the presence of biologically significant stimuli, such as threats, demonstrates our adaptive ability to maximize survival prospects in our environment.

Biologically relevant stimuli go beyond threats: they also include stimuli crucial for survival and reproduction, such as infant faces (Brosch et al., 2007; Sakaki et al., 2012). Lorenz (1943) introduced the concept of the “baby schema,” which refers to the distinctive physical characteristics of infants that trigger positive reactions and caregiving behaviors in adults, such as large eyes, round faces, chubby cheeks, and small noses and mouths. The baby schema's evolutionary role is believed to enhance the chances of infant survival (Glocker et al., 2009; Lorenz, 1943). Neuroimaging studies have shown that infant faces activate regions associated with attention, reward, and motor more strongly than do adult faces, both in non‐parents and in parents (Bornstein et al., 2017; Caria et al., 2012; Endendijk et al., 2020; Glocker et al., 2009; Luo et al., 2015; Raghunath et al., 2022). This suggests that our brain adjusts its perception of infants to elicit positive reactions and caregiving behaviors. The question remains if the perception of infant faces also triggers distortions in time perception.

Research indicates three primary responses triggered by the babyface schema: a preference response (Franklin et al., 2018; Jia, Ding et al., 2021), an attentional bias (Brosch et al., 2007; Jia, Ding et al., 2021; Jia et al., 2022; Lucion et al., 2017), and a viewing motivation (Ding et al., 2016; Jia, Ding et al., 2021). The attention bias towards infant faces implies that adults are more drawn to these faces than to adult faces. Brosch et al. (2007) first demonstrated this bias using a dot‐probe paradigm, where participants had to locate a dot after being presented with a neutral infant face and an adult face. Their results indicated that adults showed a stronger attention bias towards infant faces in comparison to adult faces. Jia and colleagues (2021) not only confirmed these findings but also found that neutral infant faces garnered the most attention compared with sad and happy expressions. They theorized that adults focus on neutral infant faces to assess infants' comfort levels. Yet, most studies only evaluate attention bias post the image display, overlooking perception during viewing. Furthermore, the impact of varying infant expressions (e.g., neutral vs. sad) on other perception aspects, such as timing, remains underexplored. Therefore, this study seeks to understand adults’ perception of time while they are observing infant and adult facial expressions. Considering that neutral and emotional infant faces may elicit different reactions from adults (Jia, Ding et al., 2021; Jia et al., 2022), we also compared the babyface schema effects in temporal perception between neutral and emotional facial expressions.

Earlier research suggests that facial features, especially attractiveness, can alter our sense of time (Ogden, 2013; Tian et al., 2019; Zhou et al., 2021). For instance, Ogden (2013) found that when women verbally estimated the duration that they viewed attractive, neutral, and unattractive female faces, they perceived both attractive and neutral faces as lasting longer than unattractive ones. Extending on this, Tian et al. (2019) found that attractive faces, regardless of sex, appeared to last longer for women. In contrast, men's perception was notably influenced by the attractiveness of opposite‐sex faces more than of same‐sex faces. Additionally, Zhou et al. (2021) highlighted the mediation role of arousal between attractiveness and time distortion. Essentially, heightened arousal from viewing attractive faces extended the perceived viewing time, while dampened arousal reduced this effect. These findings underscore how our sense of time might be interwoven with facial characteristics. Furthermore, participants' sex could influence how facial characteristics affect their time perception. Notably, the impact of sex on attention bias induced by babyface schema has yielded mixed findings. Some research suggests that women display a more pronounced attentional bias to infant faces than men do (Cárdenas et al., 2013; Colasante et al., 2017; Jia, Ding et al., 2021; Zhang et al., 2022), while other studies report little to no difference between men and women (Brosch et al., 2007; Ding et al., 2016; Jia et al., 2022; Long et al., 2021). The variation in results across these studies could stem from the use of different experimental paradigms. Therefore, it is essential to consider sex when studying time perception in relation to the babyface schema.

Within time perception studies, researchers use two primary theories to explain time distortion by emotional stimuli: the internal clock with the pacemaker–accumulator perspective (Gibbon, 1977; Gibbon et al., 1984; Gibbon & Church, 1990) and the concept of embodied timing (Droit‐Volet et al., 2013; Wittmann, 2013, 2014). The internal clock model posits a pacemaker–accumulator mechanism, with the pacemaker and accumulator connected by a switch. As stimulus timing commences, this switch activates, allowing the pacemaker to send time‐representative pulses to the accumulator. Distortions in time perception, according to this model, arise from variations in the pacemaker rate and the switch operation. Stimuli of high arousal may accelerate the pacemaker, leading to a denser stream of pulses over time and, consequently, to an extended subjective time perception. Such a phenomenon is evident in studies involving emotionally charged perceptions such as anger, threats, and attractiveness, underscoring the pivotal role of arousal (Angrilli et al., 1997; Droit‐Volet et al., 2004; Droit‐Volet & Berthon, 2017; Droit‐Volet & Dambrun, 2019; Gil & Droit‐Volet, 2012; Tian et al., 2019; Zhou et al., 2021). Conversely, highly salient stimuli can trigger an earlier switch activation, enabling more temporal pulses to pass through and be recorded (Grommet et al., 2011; Lui et al., 2011). Additionally, Zakay and Block (1997) further included attention in the model, with an attentional gate situated between the pacemaker and the switch. This gate dictates attention directed towards the timing process. When it is more open, more pulses are conveyed to our cognitive counters. If timing garners more attention, this gate will widen and let more pulses through, leading to extended subjective time. Based on this theory, Ogden (2013) argued that less attractive faces divert attention from timing, resulting in a reduction of time estimation compared with their attractive and neutral counterparts.

Building on the idea that our perception of time is intertwined with our bodily state, the theory of embodied timing (Wittmann, 2014; Wittmann et al., 2010; Wittmann & van Wassenhove, 2009), rooted in embodied cognition (Clark, 1999; Engel et al., 2013; Niedenthal, 2007), posits that our experience of time not only is a perceptual process, but also is shaped by the dynamic interplay between our body and its surrounding context (Droit‐Volet, 2019; Droit‐Volet et al., 2013). For instance, facing a threat causes our bodies to brace for potential defensive actions. This can speed up somatosensory processing, gearing us up for swift and accurate reactions (Droit‐Volet et al., 2013; Gil & Droit‐Volet, 2011; Jia et al., 2015; Shi et al., 2012). A recent comprehensive meta‐analysis of 95 studies on time perception suggested that the activation of pre‐supplementary motor area (SMA) and the bilateral insula during timing context are associated with the sensorimotor and interoceptive views of embodied timing (Naghibi et al., 2024). Furthermore, some researchers have blended the pacemaker–accumulator and the embodied timing theories to shed light on time distortion (Pollatos et al., 2014; Zhou et al., 2021). For instance, Zhou et al. (2021) not only highlighted the crucial role of arousal in time perception, but aslo hinted that appealing adult faces could evoke approach motives (Rhodes, 2006). This either boosts arousal or captures attention, influencing perceived duration.

Drawing from prior research and the theoretical frameworks discussed, this study aims to investigate the influence of babyface schema on duration perception. Specifically, we intend to address the following three primary research questions. (1) Does the babyface schema influence temporal perception when participants encounter both infant and adult facial expressions? (2) Are neutral and emotional infant facial expressions equivalent in their effects on time perception? This study employs crying infant expressions as emotional stimuli, given that crying is an infant's primary mode of communicating with adults. It evokes an adult's reaction and caregiving behavior, which are essential for the infant's survival (Bornstein et al., 2017; Brosch et al., 2007; Lorenz, 1943; Soltis, 2004; Young et al., 2016). Anchored in the embodied timing perspective, the way that adults perceive time can assist them in gauging a situation's urgency, prompting them to swiftly aid distressed infants. (3) Does the participant's sex sway the impact of the babyface schema on perceived duration? While empirical studies (Brosch et al., 2007; Cárdenas et al., 2013; Colasante et al., 2017; Ding et al., 2016; Jia, Ding et al., 2021; Jia et al., 2022; Long et al., 2021; Zhang et al., 2022) on the modulation of sex in attention bias towards babyface schema have shown inconsistent results, Tian et al.'s (2019) investigation of the influence of facial characteristics in time perception revealed sex differences. Given these varying findings, sex was included as a variable in this study.

To address these research questions, two experiments were designed. Experiment 1 adopted neutral infant expressions, while Experiment 2 integrated sad (crying) infant expressions. Furthermore, we assessed sex‐based differences in duration judgments for both infant and adult facial expressions across the two experiments.

## EXPERIMENT 1

### Methods

#### Participants

A total of 56 university students (28 females and 28 males; *M* = 20.55 years) participated in the experiment. The sample size was larger than that of a previous similar study (40 participants; Tian et al., 2019). A priori power analysis indicated that a sample size of 44 would provide sufficient power (1 – β ≥ 0.95) to detect a medium effect (η_*p*_^*2*^ = 0.06) (Faul et al., 2007). All participants were unmarried and childless Chinese adults with normal or corrected‐to‐normal vision. Before the experiment, they signed an informed consent form. The experiment was approved by the Ethics Committee of Jiangnan University.

#### Stimuli and apparatus

Participants undertook the experiment in a dim, quiet room. They viewed stimuli on a 24‐in. LCD monitor with a resolution of 1024 × 867 pixels and a 100‐Hz refresh rate. A chin rest ensured that they kept a consistent 57‐cm viewing distance. The presentation of stimuli and data collection were coded in Matlab 2018b (MathWorks, Natick, USA), employing the PsychToolbox Version 3 (Brainard, 1997; http://psychtoolbox.org). From the Chinese multi‐expression database for infants and adults (Jia et al., 2019), we selected 12 neutral facial expression images as target stimuli. Half portrayed infants (four females, two males), while the other half depicted adults (three females, three males). Each facial image measured 260 × 300 pixels, set against a gray background.

Before the main experiment, 18 other individuals rated the arousal and valence of the infant and adult faces using a 9‐point Likert scale (Bradley & Lang, 1994). Neither arousal (infant: *M* = 5.70 vs. adult: *M* = 5.22) nor valence (infant: *M* = 5.22 vs. adult: *M* = 4.57) displayed a significant difference between the two face categories (both *p*s *> *.05).

#### Procedure

The experiment used a temporal bisection task. To familiarize participants with both short and long references, they viewed a white square (5.3° × 5.3° visual angle) for two standard durations – one short (300 ms) and one long (900 ms). Each duration appeared five times. Subsequently, participants had to categorize durations as “short” or “long” using designated keyboard keys for 20 trials (10 of which were short and 10 of which were long). At the end of the practice test, the accuracy rate was displayed on the screen. The formal experiment started only after participants achieved a 95% or higher accuracy in judging the standard durations. If a participant failed to reach the required accuracy, they received another 20 trials. All participants passed the practice test within 40 trials.

**FIGURE 1:**
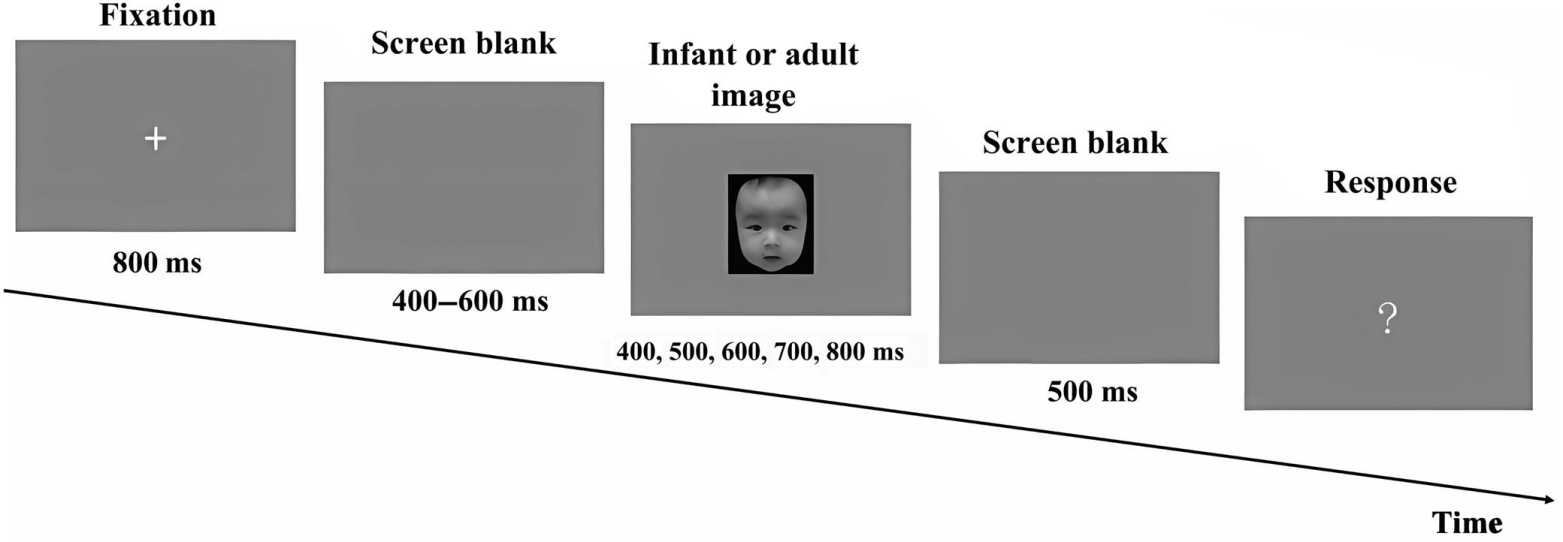
Schematic illustration of stimuli presentation in Experiment 1. Each trial began with a fixation cross, followed by a blank screen lasting from 400 to 600 ms. Subsequently, a neutral infant or adult face appeared for one of five varying probe durations. After a blank screen lasting for 500 ms, a question mask was displayed, prompting participants to judge whether the probe duration was closer to the short or the long standard duration.

Figure 1 illustrates a trial's structure for the formal experiment. The trial began with an 800‐ms white fixation point (“+”), followed by a blank screen, lasting for 400 to 600 ms. A facial image, of either an infant or an adult, was then displayed for a randomly chosen probe duration (options: 400, 500, 600, 700, and 800 ms). The set of durations was selected based on past research for better comparison (duration selection referenced from Droit‐Volet et al., 2010; Jia, et al., 2021; Shi et al., 2012). A 500‐ms blank screen ensued, and then a white question mark appeared. Participants then decided whether the probe duration was closer to the short or the long standard, and pressed the respective key (Up arrow key for “short” and Down arrow key for “long”). Each trial had a 2000‐ms inter‐trial interval.

In the main experimental phase, participants first completed two practice blocks, each with 15 trials. They then moved on to six experimental blocks, each comprising 30 trials. Each experimental condition (Face category × Durations) was repeated 18 times and randomly tested on a trial‐by‐trial basis.

### Results

**FIGURE 2:**
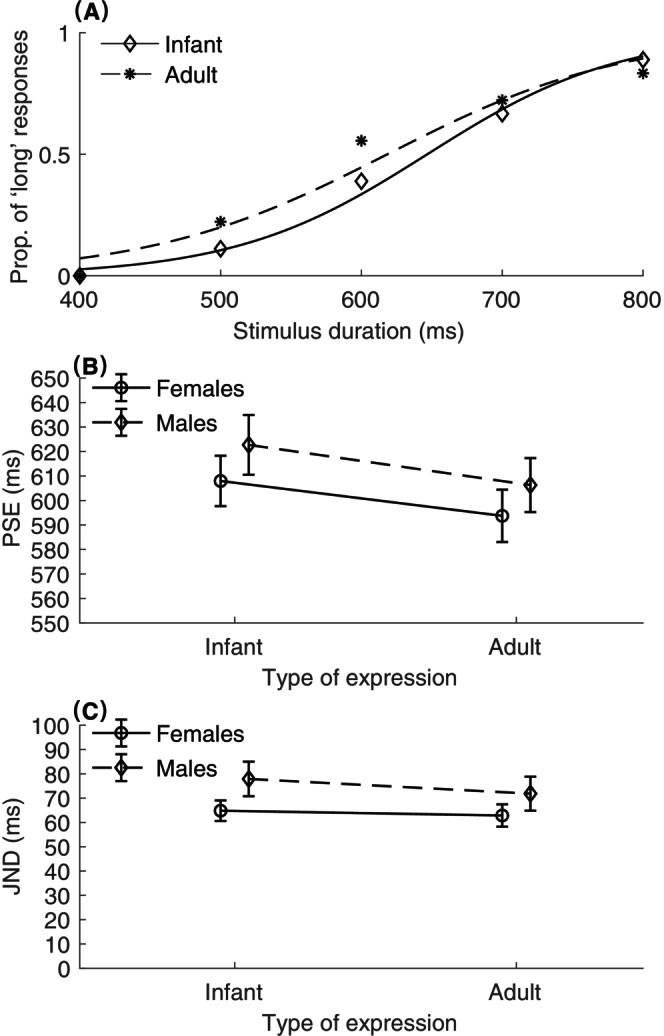
Results of Experiment 1. (A) Mean proportions of “long” responses, respectively for the infant (diamonds) and adult (stars) facial expression conditions, and their associated psychometric curves, from a representative participant. (B) The point of subjective equality (PSEs) and the corresponding standard errors are plotted against the type of expression (infant vs. adult) for female (circles) and male (diamonds) conditions. (C) Mean just‐noticeable difference (JNDs) and the associated standard errors plotted against infant and adult face conditions, for females (circles) and males (diamonds).

The proportions of the “Long” judgments for each facial category and participant were modeled using a logistic function. Figure 2(A) illustrates the fitted logistic psychometric curves for the infant and adult facial categories from a representative participant. The bisection point (BP) is the probe duration at which the logistic function hits a 50% threshold, often referred to as the point of subjective equality (PSE). A lower BP indicates a tendency to perceive durations as longer. For assessing discrimination sensitivity, the just‐noticeable difference (JND) was derived. The JND represents the distance between the 25% and 75% thresholds of the fitted curve. A larger JND indicates a lower sensitivity to discerning varied durations, reflecting inferior temporal discrimination.

Figure 2(B) depicts the mean PSEs (and associated standard errors, SEs) for neutral infant and adult facial categories, separated for male and female participants. The PSEs (± SEs) were 608 ± 10.10 (infant/female), 594 ± 10.49 (adult/female), 623 ± 11.98 (infant/male), and 606 ± 10.82 ms (adult/male), respectively. We conducted mixed repeated‐measures analyses of variance (ANOVAs) for PSEs and JNDs, designating expression type (infant vs. adult) as a within‐subject factor and participant sex (female vs. male) as a between‐subject factor. The analysis showed that mean PSEs were significantly higher in the infant condition (*M* = 615 ms, *SE* = 7.83 ms) than in the adult condition (*M* = 600 ms, *SE* = 7.52 ms), *F*(1,54) = 13.92, *p < *.001, η_p_^2^ = 0.21, suggesting that participants felt that the infants’ faces lasted for a shorter duration. Neither the main effect of sex nor the interaction of expression type with sex showed a significant difference (Sex: *F*(1,54) = 0.85, *p = *.36, η_p_^2^ = 0.02; Interaction: *F*(1,54) = 0.07, *p = *.79, η_p_^2^ = 0.001).

Figure 2(C) describes the JNDs (and SEs) for neutral infant and adult facial categories, separated for male and female participants. For JNDs, the ANOVA did not yield significant findings for the main factors of sex and expression type, or for their interaction (*F*(1,54)s < 2.08, *p*s > .16, η_p_^2^ s < 0.04).

In our first experiment, we compared neutral infant faces with neutral adult faces to examine the impact of the babyface schema on temporal perception and found that participants, regardless of their sex, viewed the duration of neutral infant faces as shorter than that of neutral adult faces. This raised a question: Would the emotional babyface schema – especially crying – affect men's and women's time perception differently? This is particularly intriguing considering women's pivotal role in childcare, and recent studies have shown a sex difference in time perception (Arantes et al., 2021; Glicksohn & Hadad, 2012; Tian et al., 2019). To investigate this, we designed a second experiment mirroring Experiment 1, but this time focusing on the facial expressions of crying infants and crying adults.

## EXPERIMENT 2

### Methods

#### Participants

A total of 56 Chinese university students (28 females and 28 males; *M* = 21.07 years) participated in this experiment. The sample size mirrored that of Experiment 1 to ensure comparability. None of the participants were married or had children. All had normal or corrected‐to‐normal vision and gave informed consent before the experiment. The experiment was approved by the Ethics Committee of Jiangnan University.

#### Stimuli and procedure

The experimental design and procedure closely resembled those for Experiment 1, except that this experiment featured crying facial expressions instead of the neutral ones from before. Specifically, we chose six crying images for both the infant and the adult faces.

Again, 18 other individuals rated the arousal and valence of the images on a 9‐point Likert scale. Neither arousal (Infant: *M* = 6.65 vs. adult: *M* = 6.25) nor valence (Infant: *M* = 4.09 vs. adult: *M* = 3.81) ratings differed between the two face categories (both *p*s *> *.05). The *t* tests indicated that the crying infant faces used in this experiment were perceived as more arousing and more negative than the neutral infant faces employed in Experiment 1 (valence: *p = *.001; arousal: *p < *.01). Similarly, significant differences were observed in the subjective ratings of valence and arousal levels between neutral and crying adult faces (valence: *p = *.05; arousal: *p < *.01).

### Results

**FIGURE 3:**
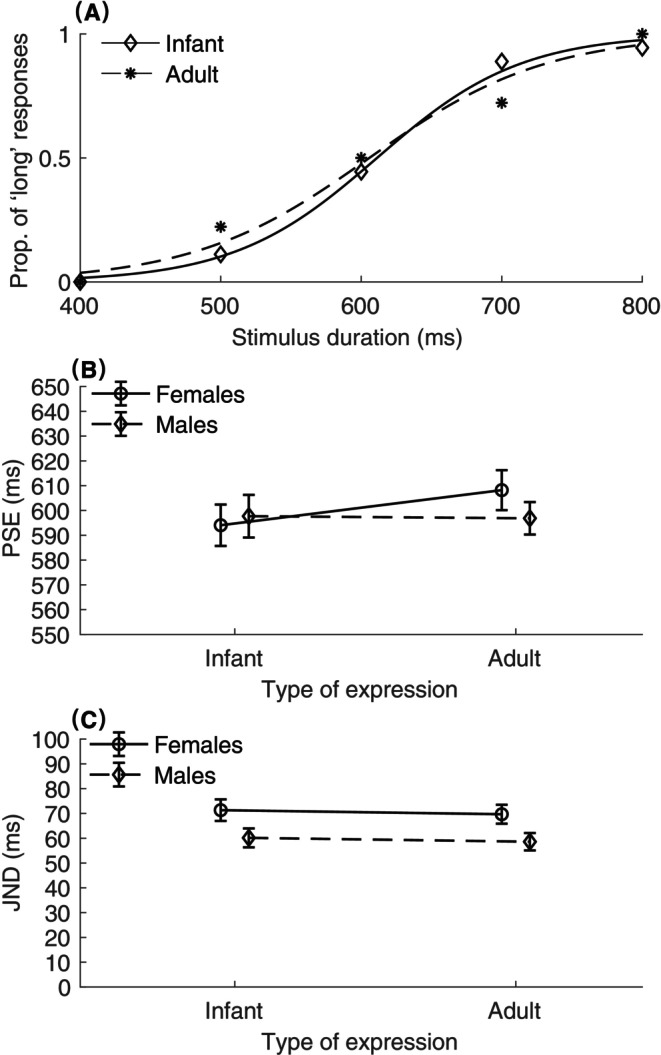
Results of Experiment 2. (A) The mean proportions of “long” responses are presented for both infant (diamonds) and adult (stars) facial expression conditions, and their associated psychometric curves, obtained from a representative participant. (B) The point of subjective equality (PSEs) and the corresponding standard errors are plotted against the type of expression (infant vs. adult) for female (circles) and male (diamonds) conditions. The two lines cross each other. (C) Mean just‐noticeable difference (JNDs) and the associated standard errors plotted against infant and adult face conditions, for females (circles) and males (diamonds).

Figure 3 presents the average PSEs and JNDs across the experimental conditions. A two‐way ANOVA was conducted on PSEs, with the factors of expression type (infant vs. adult) and participant sex (female vs. male), which revealed a marginal significant interaction between expression type and participant sex, *F*(1,54) = 3.65, *p = *.06, η_p_^2^ = 0.06. However, neither the expression type nor the participants' sex showed a significant effect [expression type, *F*(1,54) = 2.87, *p = *.10, η_p_^2^ = 0.05; sex, *F*(1,54) = 0.14, *p = *.71, η_p_^2^ = 0.003]. The interaction was primarily contributed by differential patterns exhibited by the male and female participants. Post hoc tests with Holm–Bonferroni‐corrected comparisons revealed a significant difference in PSEs for female participants, with the average PSE for infant expressions (*M* = 594 ms, *SE* = 8.21 ms) being significantly lower than that for adult expressions (*M* = 608 ms, *SE* = 7.90 ms), corrected *p < *.05. This suggests that female participants felt that the crying‐infant images lasted longer than the crying‐adult images. In contrast, for male participants, no significant PSE variation was found between infant (*M* = 598 ms, *SE* = 8.45 ms) and adult (*M* = 597, *SE* = 6.44 ms) expressions, corrected *p = *.88.

In the case of JNDs, a two‐way ANOVA indicated that male participants had a significant reduction of the JND (*M* = 59 ms, *SE* = 2.52) in comparison to females (*M* = 71 ms, *SE* = 2.82), *F*(1,54) = 5.55, *p < *.05, η_p_^2^ = 0.10. This suggests that temporal judgments were more sensitive to emotional stimuli in females than in males. However, neither the main effect of expression type nor the interaction was significant [expression type, *F*(1,54) = 0.38, *p = *.54, η_p_^2^ = 0.01; interaction, *F*(1,54) = 0.00, *p = *.99, η_p_^2^ = 0.00].

## DISCUSSION

This study aimed to investigate how infant facial expressions influence perceived duration. Using neutral facial expressions, we found that participants consistently felt that neutral infant faces lasted for a shorter duration than did neutral adult faces, regardless of their sex. However, when we tested crying facial expressions, we observed that women perceived crying infant faces as lasting for longer than crying adult faces, while men did not show any significant difference between the two. These results underscore the influence of the babyface schema on how we perceive time, notably the varying impacts between neutral and crying expressions.

Infant facial expressions, being potential attention‐grabbers, have been shown to lead to biases in spatial attention compared with adult facial expressions in the dot‐probe paradigm (Jia, Ding et al., 2021; Jia et al., 2022; Lucion et al., 2017). The common explanation, according to the pacemaker–accumulator account, is that this attentional pull from infants’ faces can cause an earlier closure of the switch (Zakay & Block, 1995, 1996), thereby accumulating more temporal pulses and lengthening the perceived duration (Gibbon, 1977; Gibbon et al., 1984; Gibbon & Church, 1990; Thomas & Weaver, 1975; Wearden et al., 1998). However, Experiment 1 of our study revealed an opposite trend: neutral infant faces seemed to compress rather than extend the perceived duration. It is worth noting that the comparison durations we used spanned between 400 and 800 ms, which surpasses the typical durations (usually up to 240 ms) seen in studies focusing on attentional biases arising from infant faces (Lucion et al., 2017). A recent study found that the attentional preference for infant faces vanished when faces appeared for 500 ms in a dot‐probe task (Long et al., 2021). This suggests that the lack of influence from attentional capture on time perception in Experiment 1 may be partially attributed to the short‐lived nature of attention capture owing to the brief stimulus presentation.

The brief window of attention capture towards infant faces aligns with the assumption that attention bias operates via an early pre‐attentive and a later attentive process (Posner et al., 1987; Theeuwes, 2010). The early pre‐attentive process is an instinctive, bottom‐up, salience‐driven process, independent of the observers' intention. In contrast, the attentive process unfolds more gradually and is influenced by top‐down control. In this context, neutral infant faces, which signal survival but radiate ambiguous emotions, swiftly capture an observer's attention so that the infant's comfort can be gauged (Jia, Ding et al., 2021). The initial attentional capture likely caused an overestimation of time for emotional stimuli, as observed in prior research (Grommet et al., 2011; Lui et al., 2011). However, when the neutral infant image is continuously focused on, the extended stimulus display might have heightened the attentive process to decode the infant's ambiguous neutral expression (non‐temporal process) rather than to monitoring the timing process. This divided attention, according to the attention gate model (Zakay & Block, 1997), could be a potential factor that impacted the shortened perceived duration we observed in Experiment 1.

While Experiment 1 showed a subjectively perceived shortened duration for neutral infant faces, Experiment 2 highlighted that women felt that crying infant faces lasted for longer than crying adult faces. Crying infant faces, in stark contrast to crying adult ones, distinctly convey distress and a palpable need for consolation and safeguarding. Such expressions in infants can trigger an adult's caregiving behavior (Bornstein et al., 2017; Bowlby, 1969; Soltis, 2004). The extended perceived duration for crying infant faces among females might reflect their heightened responsiveness and inclination to comfort and care for the upset infant. This interpretation aligns with the embodied timing theory, which posits that our sense of time is closely linked to our perception–action loop (Clark, 1999; Droit‐Volet et al., 2013; Engel et al., 2013; Wittmann, 2014). Previous studies have demonstrated that emotional stimuli, particularly those prompting imminent actions, can lead to a subjective expansion of time (Droit‐Volet, 2019; Droit‐Volet & Gil, 2009; Droit‐Volet & Meck, 2007; Gil & Droit‐Volet, 2011; Jia et al., 2015). Within the context of our study, the heightened urge to provide prompt care and support for crying infants may lead to an acceleration of the sensory processing (Craig, 2009; Hagura et al., 2012), thereby stretching the perceived duration.

One may question if arousal influences the duration overestimation of crying infant faces. In our study, a separate assessment had participants rate the arousal of both crying infant and crying adult faces, which revealed no significant difference between these facial expressions. Moreover, despite the equivalent arousal levels between crying infant and crying adult faces, only the females felt that images of crying infants lasted longer than those of crying adults. Therefore, arousal, if there is any, is not the primary factor driving the perceived duration expansion in Experiment 2. On the other hand, we cannot fully rule out the potential influence of attentional capture. Given the clear signal of an urgent need for caregiving behavior, an infant's crying expression could sustain observers' attention to it, leading to an earlier closure of the switch and the accumulation of more temporal pulses, thus expanding the image's subjective duration. However, a recent study by Jia, Ding et al. (2021) revealed only a weak effect of attention bias for sad faces compared with neutral ones. Additionally, the expressions of crying in adults might be perceived as more intricate and unclear compared with those of infants (Vingerhoets & Bylsma, 2016). The crying of adults may be triggered by conflict, loss, failure, weddings, or reunions. Echoing the findings of Experiment 1, the ambiguous nature of adult crying expressions might shift attention away from monitoring the timing, resulting in a perceived reduction in the duration of adult crying images. The present study was unable to clearly differentiate between these two interpretations, given that the bisection task measured the relative perceptions of short or long durations. Alternative methodologies, such as duration reproduction, could be used in future studies to clarify these points. Nevertheless, both experiments highlight the likely influence of attention on subjective time distortion. When attention is sidetracked from the timing process owing to ambiguity, durations tend to be perceived as shorter. Conversely, a distinct and urgent cue that commands attention can lead to duration overestimation.

On another note, the outcomes from the two experiments differed in sex‐specific patterns. In Experiment 1, both female and male participants displayed a reduced perceived duration for neutral infant faces compared with neutral adult faces. This aligns with findings from various dot‐probe paradigm studies (Brosch et al., 2007; Ding et al., 2016; Jia et al., 2022; Parsons et al., 2011). In contrast, Experiment 2 highlighted a sex‐differentiated impact on perceived durations for crying infant faces. Notably, female participants felt that there was a longer duration when watching crying infants compared with when watching crying adults, a distinction not evident in male participants. Using the lens of embodied timing, the expanded perceived duration in females suggests a heightened instinctual drive in them to nurture and shield crying infants. This inclination finds backing in neurobiological research, which indicates that sad infant faces trigger more potent neural activations in behavior‐relevant brain regions in females than in males (Messina et al., 2016; Zhang et al., 2022). More research is essential to explore further the mechanisms and potential neural correlates underpinning these sex‐based discrepancies in time perception.

While our study serves as a pioneering exploration into how infant faces influence time perception, it also opens several avenues for future research. This initial investigation lays the groundwork for more comprehensive studies, highlighting areas for further exploration and validation. For instance, our focus on sub‐second durations invites subsequent research to examine a broader range of durations, including super‐seconds. This would help clarify the distinct contributions of attention (additive effect) and arousal (multiplicative effect) to how we perceive time in response to infant facial expressions (Burle & Casini, 2001; Droit‐Volet & Meck, 2007; Maricq et al., 1981; Penton‐Voak et al., 1996). Additionally, incorporating assessments of arousal for each facial image could reveal correlations between subjective duration and arousal levels, enriching our understanding of these dynamics. Third, exploring whether the duration effect we observed with infant faces extends to other “small” stimuli, such as children versus adults, or puppies versus adult dogs, could broaden the applicability of our findings. Furthermore, considering participant characteristics, such as their desire for or familiarity with children, may shed light on individual differences in time perception. Together, these directions not only address the limitations of our initial study but also promise to deepen our understanding of time perception.

In conclusion, our study extends the understanding of the influence of the babyface schema on time perception. Neutral infant faces, owing to their ambiguity, led to a shortened perceived duration, while crying infant faces lengthened perceived duration for females, lending weight to the concept of embodied timing.

## CONFLICT OF INTEREST STATEMENT

The authors declared no potential conflicts of interest with respect to the research, authorship, and/or publication of this article.

## ETHICS STATEMENT

The study was approved by the Ethical Committee of Jiangnan University, in accordance with the Declaration of Helsinki.
